# Implementing a global approach for efficiently simulating molecular dynamics in agent-based models of biological tissue

**DOI:** 10.1016/j.xpro.2022.101777

**Published:** 2022-10-25

**Authors:** Daniel Bergman, Trachette L. Jackson

**Affiliations:** 1 Department of Mathematics, University of Michigan, Ann Arbor, MI 48109, USA

**Keywords:** Systems biology, Computer sciences

## Abstract

This protocol explains how to take an agent-based model (ABM) with molecular dynamics and set it up to solve the molecular dynamics with a global approach. It can be used to speed up simulations significantly while retaining high levels of accuracy with the original ABM. Two options are presented for implementing this global approach, depending on the desired spatial variability in molecular concentrations. Both options coarse-grain the molecular dynamics in space by dividing the microenvironment into regions with uniform concentrations.

For complete details on the use and execution of this protocol, please refer to Bergman et al. (2022).

## Highlights

• Efficiently solve molecular dynamics in agent-based models of biological tissue
• Coarse-grain microenvironment into uniform regions to reduce complexity
• Use average intracellular dynamics for all cells to increase efficiency
• Implement in an ABM to speed up exploration and analysis

## Abstract (editor highlights)

Publisher’s note: Undertaking any experimental protocol requires adherence to local institutional guidelines for laboratory safety and ethics.

## Graphical abstract

**Figure:**
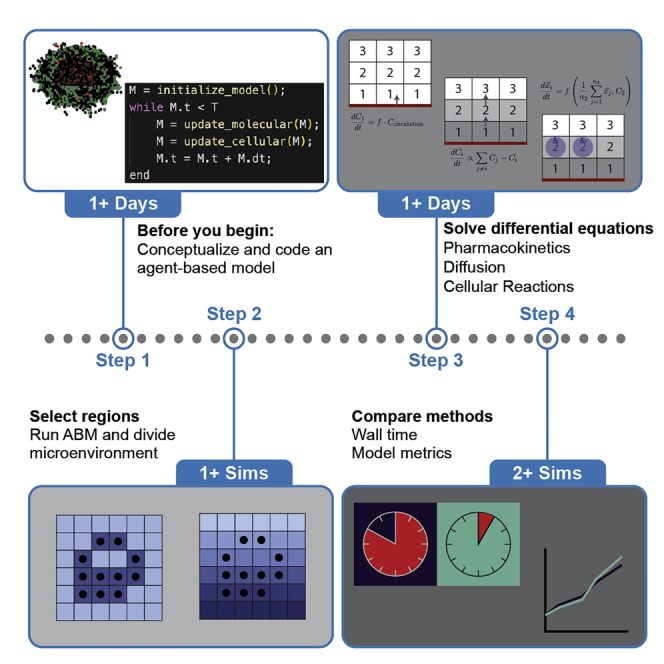

## Before you begin

The protocol below describes setting up a global method for solving molecular dynamics in an on-lattice ABM. Before beginning the protocol, you must first set up this ABM. We outline here how such an ABM could be set up, but full implementation details are beyond the scope of this protocol.

### Institutional permissions

None.

### Download MATLAB

**Timing: 1 h**

1. Download and install MATLAB (https://www.mathworks.com/help/install/install-products.html).

***Note:*** Code will be provided in MATLAB syntax. Those familiar with other coding languages--such as C++, Python, and Julia—should find it straightforward to implement this in their preferred language.

***Note:*** No additional packages are necessary to make the method work.

### Conceptualize the ABM

**Timing: 1–4 h**

Fully describing an ABM is beyond the scope of this protocol. Here, we outline some typical components of an ABM and decisions a modeler must make in setting these up.

2. Determine how cells update in the model.
  a. Proliferation.
    i. Where to place the new cell and what happens if that lattice site is occupied?
    ii. Symmetric or asymmetric?
    iii. Include multiple phases of cell cycle?
  b. Apoptosis.
    i. How long until apoptotic cells are removed from the lattice?
  c. Movement.
    i. What happens when the cells is at the boundary of the lattice?
    ii. How do neighboring cells affect cell movement?
    iii. Moore or Neumann neighborhood?
    iv. Chemotaxis?
  d. Etc.
3. Determine how the molecular agents interact with the cells, answering the following questions.
  a. How do the molecules enter the environment (extravasation, secretion, etc.)?
  b. How do cells interact with the molecule (sample local concentration, react with surface receptors, uptake, etc.)?
  c. How are cells affected by the molecule (changes in cell cycle, changes in behavior, etc.)?

### Code the skeleton of the ABM

**Timing: 2 h to 1+ days**

4. Initialize cells on a lattice.
  a. Decide on lattice size.
  b. Choose method for placing initial cells.
    i. Uniform random across all lattice sites.
    ii. Near the center of the mesh.
    iii. Other.
5. Set update functions for cells.
  a. Proliferation.
  b. Apoptosis.
  c. Movement.
  d. Etc.

### Code molecular dynamics

**Timing: 2 h to 1+ days**

6. Initialize molecule.
  a. Set concentration at each lattice site.
  b. Specify blood vessel locations, if applicable.
7. Set up molecular dynamics solver.
  a. Pharmacokinetics (PK).
  b. Diffusion dynamics.
  c. Cellular-molecular reactions.
8. Link molecular state to cellular state.
  a. Update proliferation, etc. rates.
  b. Update symmetric division probability.
  c. Etc.

## Key resources table

**Table:** 

REAGENT or RESOURCE	SOURCE	IDENTIFIER
**Software and algorithms**		

MATLAB	MathWorks, Inc.	https://www.mathworks.com/products/matlab.html
FGFR3 and IL-6 models	Bergman et al. (2022); Zenodo	https://doi.org/10.5281/zenodo.6470845

## Step-by-step method details

For setting up the global method, two options are: 1) divide the microenvironment into occupied and unoccupied regions or 2) partition the microenvironment into several fixed regions. Both options were demonstrated in in Bergman et al. (2022) to simulate a fibroblast growth factor receptor 3 (FGFR3) model and an interleukin-6 (IL-6) model, respectively. Links to the relevant lines in the GitHub repository are provided throughout. These two models involve a drug diffusing in the microenvironment of a tissue reacting with cell surface receptors to alter cell fate decisions. After setting up the ABM and before implementing the global method, see if the variability in molecular states among cells when making cell fate decisions is large enough that it ought to be captured in the model. If not, use Option 1. If there is such large variability, look for spatial dependence of this variability and use that to inform a choice or regions for Option 2.

### Create ODEs for molecular dynamics

**Timing: 1 day**

**Figure 1:**
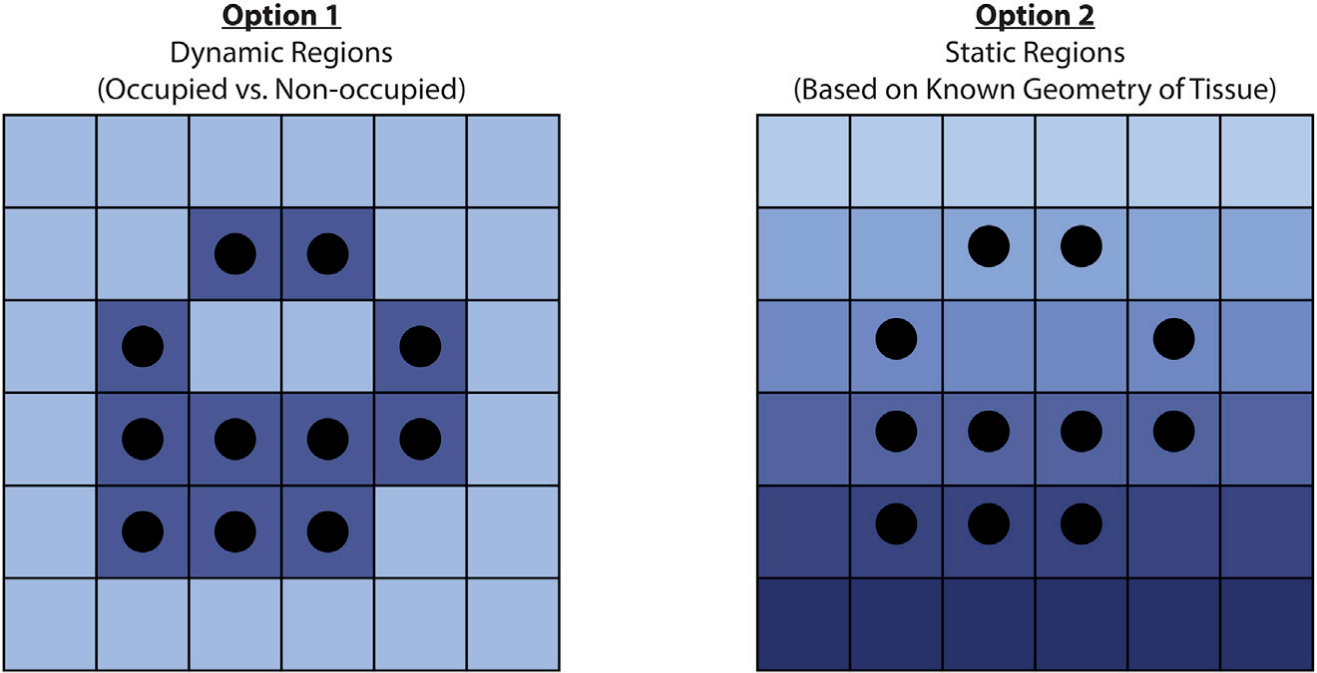
Illustrative examples of the two options for regions Adapted with permission from Figure 1 of Bergman et al. (2022).

Two options are presented here: 1) divide the microenvironment into occupied and unoccupied regions or 2) divide the microenvironment into fixed regions, such as by distance from vasculature (see Figure 1). If all cells in the original ABM are affected similarly by the molecular dynamics, Option 1 is appropriate. If the original ABM shows clear spatial effects of the molecular dynamics on the cellular dynamics, strongly consider using Option 2. The code here outlines how to build a single ordinary differential equation (ODE) that then captures the three differential equations—pharmacokinetics (PK), diffusion, and cellular reactions. In Bergman et al. (2022), we solve these equations in a single system of ODEs by adding up how each state variable changes in the differential equations describing the key molecular dynamics.

#### Option 1: Occupied and unoccupied regions

This option divides the microenvironment into two regions: one occupied by cellular agents and the other unoccupied by cellular agents. As cells proliferate, die, and move, these regions will change. Molecular states are averaged within each region to solve the molecular dynamics.

1. Replace concentration variable for the diffusing molecule with two variables: the occupied region concentration and the unoccupied region concentration.
2. PK dynamics, if applicable.
  a. The concentration in the two regions changes based on the proportion of those regions that contain blood vessels. See here.

>% C_occupied = concentration at occupied lattice sites

>% C_unoccupied = concentration at unoccupied lattice sites

>% p_occupied = proportion of occupied region near blood vessel

>% p_unoccupied = proportion of unoccupied region near blood vessel

>% exchange_rate = rate of extravasation into microenvironment

>% C_circ = concentration in circulation

>dC_occupied_dt = p_occupied ∗ exchange_rate ∗ C_circ;

>dC_unoccupied_dt = p_unoccupied ∗ exchange_rate ∗ C_circ;

***Note:*** If your ABM allows for the substrate to diffuse back across the capillary walls based on the concentration differential, use the following instead:

>dC_occupied_dt = p_occupied ∗ exchange_rate ∗ (C_circ – C_occupied);

And likewise for the unoccupied concentration.

3. Diffusion dynamics.
  a. Diffusion depends on the proportion of the microenvironment each region compromises. See here.

>% q_occupied = proportion of microenvironment that is occupied

>% q_unoccupied = proportion of microenvironment that is unoccupied

>% D = diffusion coefficient

>% h = distance between lattice sites

>dC_occupied_dt = 6 ∗ D ∗ q_unoccupied ∗ (C_unoccupied – C_occupied) / hˆ2;

>dC_unoccupied_dt = 6 ∗ D ∗ q_occupied ∗ (C_occupied – C_unoccupied) / hˆ2;

***Note:*** The factor 6 can be understood as 6 = 2 × (number of dimensions). Thus, for a 2D simulation, replace 6 with 4. See here.

***Note:*** Division by hˆ2 above is due to diffusion being based on a second-order spatial derivative, not the dimensionality of the simulation.

***Note:*** If degradation of the substrate occurs in the microenvironment, this is a good place to include that. Subtract degradation_rate ∗ C_occupiedfrom the first and subtract degradation_rate ∗ C_unoccupied from the second.

***Note:*** Since the method retains full spatial resolution for the cells, at least up to the lattice, it is possible to use this information to arrive at a better coarse-graining of the diffusion between these two regions. Specifically, the value of q_unoccupied can be computed by looking at the actual proportion of lattice sites neighboring cells that are unoccupied rather than estimating this as the proportion of all sites that are unoccupied. Similarly for computing q_occupied. See the diffusion step in Option 2 below. Using this in Option 1 does require recomputing those proportions before each time solving the diffusion equation.

4. Reaction dynamics.
  a. Average across all cells and use that as initial condition for the single agent reaction equation. See here.

>% A = Nxr array of N agents and the r molecular state variables

>A_avg = mean(A,1); % average across all agents

>A_avg = A_avg'; % transpose to make it a column vector

>sol = ode45(@(t,x) reaction_ode(t,x,pars),[0 dt],A_avg); % solve

>A = repmat(sol.y(:,end)',[N,1]); % update all molecular concentrations

5. Cell fate effects on concentration.
  a. Proliferation: update the occupied concentration.
  b. Apoptosis: update the unoccupied concentration.
  c. Movement: update both the occupied and unoccupied concentrations.
  d. Use the weighted average to update the concentration(s) named above. See here.

>% N = number of agents

>% V = volume of microenvironment (in lattice sites)

>%% when a cell proliferates…

>C_occupied = (N ∗ C_occupied + C_unoccupied) / (N+1);

>%% when a cell dies…

>C_unoccupied = ((V-N) ∗ C_unoccupied + C_occupied) / (V-N+1);

>%% when a cell moves…

>C_occupied_old = C_occupied;

>C_occupied = ((N-1) ∗ C_occupied + C_unoccupied) / N;

>C_unoccupied = ((V-N-1) ∗ C_unoccupied + C_occupied_old) / (V-N);

#### Option 2: Fixed regions

This option divides the microenvironment into fixed regions. These are best chosen based on known geometric features of the distribution of the molecule. For example, based on proximity to vaculature. There can be as many regions as deemed necessary, but more regions requires more computational time. As cells proliferate, die, and move, these regions do not change, but the density of agents within each does change. Molecular states are averaged within each region to solve the molecular dynamics.

6. Divide the microenvironment into n regions, for example level sets of the distance to vasculature.
  a. Create a variable R where size(R) is the size of the lattice. See here.
  b. The values of R identify the region that lattice point belongs to. See here.
  c. These are indexes into the concentration array. Hence, use 1, 2, 3, …, n to identify the n regions with each region having a nonzero volume.
7. Replace concentration variable with a vector of concentrations representing the average concentration in each region. Call it C such that size(C) is [n,1], i.e., a column vector. See here.
8. PK dynamics, if applicable.
  a. The concentration in each region changes based on the proportion of the region that contain blood vessels. See here.

>% p = vector of proportions of each region containing blood vessels

>% exchange_rate = rate of extravasation into microenvironment

>% C_circ = concentration in circulation

>dC_dt = p ∗ exchange_rate ∗ C_circ;

***Note:*** If your ABM allows for the substrate to diffuse back across the capillary walls based on the concentration differential, use the following instead:

>dC_dt = p ∗ exchange_rate ∗ (C_circ – C);

***Note:*** We have found it works better to make each region entirely at a blood vessel or contain no blood vessel, i.e., p is a Boolean vector indicating whether a region is perivascular or not. If a region you want contains a mix, then one option is to split it into two regions: one with and one without vasculature.

9. Diffusion dynamics.
  a. Diffusion depends on the proportion of each region’s neighbors that are in a given region. See here.
  b. For regions i and j, let q_ij be the proportion of region i neighbors that are in region j.
    ***Note:*** By neighbors, we mean the 6 nearby lattice sites used in the standard stencil for solving a partial differential equation (PDE) in 3D.
  c. Include boundary conditions of the PDE at this step.
    i. Neumann conditions: consider neighboring sites that are off the lattice as belonging to the same region as the center point.
    ii. Dirichlet conditions: create your regions so that each region either only contains sites with Dirichlet conditions or contains no sites with Dirichlet conditions.
  d. The boundary conditions of the PDE should be included at this step.
  e. Compute this one time at the start of the simulation after the regions have been defined and save for reuse throughout.

>% q = nxn array where row i contains the proportions of region i neighbors in each region

>% conc_diffs = nxn array of concentration differentials; row i contains the correct sign for changing region i concentration

>% D = diffusion coefficient

>% h = distance between lattice sites

>conc_diffs = C' – C;

>dC_dt = 6 ∗ D ∗ sum(q.∗ conc_diffs,2) / hˆ2; % add along rows

>dC_unoccupied_dt = 6 ∗ D ∗ q_occupied ∗ (C_occupied – C_unoccupied) / hˆ2;

***Note:*** The factor 6 can be understood as 6 = 2 × (number of dimensions). Thus, for a 2D simulation, replace 6 with 4. See here.

***Note:*** Division by hˆ2 above is due to diffusion being based on a second-order spatial derivative, not the dimensionality of the simulation.

***Note:*** If degradation of the substrate occurs in the microenvironment, this is a good place to include that. Subtract degradation_rate ∗ C from dC_dt. See here.

10. Reaction dynamics.
  a. For each type of cell, average across all cells of that type within each region.
  b. Use this as the initial condition for the differential equation describing the reaction dynamics for a single cell of this type. See here.
  c. Must account for the concentration representing the average across the entire region.
    i. The below formulation works well if the equations are solved sequentially. That is, if these reaction equations are solved separately from the diffusion equations.
    ii. If they are solved simultaneously, then introduce a factor of p_i into the rate of change for C_i where p_i is the proportion of region i that is occupied by cells of the current type. See here. Specifically, if reaction_ode_free_i represents the rate of change for the free substrate in region i inside reaction_ode, then use.

>dC_i_dt = p_i ∗ reaction_ode_free_i(A_avg,C_i);

>% A = Nxr array of N agents in region i and the r molecular state variables

>% C_i = average concentration of substrate in region i

>% p = proportion of region that is occupied by the N agents

>A_avg = mean(A,1); % average across all agents

>A_avg = A_avg'; % transpose to make it a column vector

>sol = ode45(@(t,x) reaction_ode(t,x,pars),[0 dt],[A_avg;C_i]); % solve

>A = repmat(sol.y(1:end-1,end)',[N,1]); % update all molecular concentrations

>C_i = p ∗ sol.y(end,end) + (1-p) ∗ C_i; % update average concentration in region i by weighted average of concentration in occupied and unoccupied volumes

## Expected outcomes

The two methods, the original and the one created following the above steps, can both be run and their outputs compared. Many metrics can be used here, for example: cell counts, substrate concentration both spatial and temporal, agent locations, etc. The global method should result in a speedup over the original ABM. This speedup depends on the number of agents used and, in the case of Option 2, the number of regions. Other factors could influence the speedup such as the ODE solver used and the complexity of the cellular level dynamics, but these are not the purview of the global method.

The script Run_Figure3.m compares the two methods in the FGFR3 model found in our paper. As written, it will take several hours to run through all 40 simulations (20 of each method). You can choose to decrease nsamps and N0 to reduce the run time. You can take similar steps with the other scripts.

## Limitations

To apply the global method to other types of ABMs will require more work, particularly considering cell volume as this can vary between cells and differ from the volume of grid it resides in. The method has not yet been tailored to cases of two cell types interacting through colocalization, such as T cells recognizing antigens on another cell. In cases where the diffusion coefficient varies in space, we recommend at the moment not including the diffusion dynamics in the global method. Instead, solve the diffusion dynamics and then average afterward for the reaction equations.

## Troubleshooting

Problems arising in the implementation of this method are difficult to predict and thus difficult to prescribe a concrete set of actions without additional information about the model. We lay out here some broad principles that could apply in some situations.

### Problem 1

The global method does not produce similar population dynamics to the local method.

### Potential solution

As the global method does nothing directly to the cellular dynamics, you must first check if the two methods are producing comparable output that is then used as the input to cell fate decisions. Store and visualize the variables used in step 8 in before you begin to assess the underlying source of the problem. Then proceed to the appropriate problem below.

### Problem 2

The two methods have similar mean signal, but the local method produces much more variability than can be captured by the global method.

### Potential solution

If this variability follows a spatial pattern, use that information to set up regions as described in option 2: fixed regions. If the variability is present within regions and there is no clear subregion structure to use, then the global method might not be well-suited for you model, as of yet. If the variability can be described as deviations from a mean signal within each region, such as a normal distribution, then you could add this observed noise after step 10 in option 2: fixed regions. We have not had occasion to implement and test this method, but it is something we are considering testing.

### Problem 3

The local method produces a different mean signal than the global method.

### Potential solution

Try a smaller time step for any numerical solvers employed. If this does not resolve the issue, rerun the simulation with exactly one of the molecular dynamics still “on”. For example, turn off diffusion, degradation, and all reactions with cells by setting the relevant parameters to 0 and then see if the two methods still disagree. If so, then it is from the PK dynamics. Repeat for diffusion and cellular reactions to identify which process is causing the difference.

### Problem 4

The local method produces a different mean signal than the global method, and this arises from solving the differential equation for PK dynamics.

### Potential solution

In both options presented for the global method, it is assumed that the PK dynamics are built so that certain lattice sites exchange with the vasculature. The code presented above assumes that the substrate does not diffuse back across capillary walls into vasculature. If your original ABM does assume this, then heed the Notes after the code blocks outlining how to include PK dynamics. If your ABM instead has PK dynamics linked to Dirichlet conditions in the diffusion equation, split those off into separate region(s) as suggested in Option 2 step 9.c.ii.

### Problem 5

The local method produces a different mean signal than the global method, and this arises from solving the differential equation for diffusion.

### Potential solution

This could arise from using regions that are too large, effectively allowing substrate to instantaneously diffuse across large distances. We have found that using regions even two cells thick near blood vessels can lead to this issue when modeling an extravasating substrate. Using regions that are one cell thick, especially near the vasculature (or source of the substrate) could help.

### Problem 6

The local method produces a different mean signal than the global method, and this arises from solving the differential equation for cellular reactions.

### Potential solution

In Option 2: Fixed regions, one possible source of discrepancy here is updating the average concentration in each region after solving the reaction equation. Whichever of the two choices you select under step 10.b, make sure the proportion of that region containing agents of that type is used to update the average concentration.

In Option 1: Occupied and unoccupied regions, the cellular reaction equations should be identical, so it is possible the next problem applies.

### Problem 7

The local method produces a different mean signal than the global method, and this arises from something other than solving a differential equation.

### Potential solution

If all the above troubleshooting tips did not work, one last possible place to turn is how cell fate decisions might affect the molecular level. In particular, the method described in option 1: occupied and unoccupied regions is sensitive to when cells proliferate and die as these change the volumes that the occupied and unoccupied regions correspond to. Review step 5 to make sure the appropriate adjustments are being made as cells proliferate, die, and move.

A similar issue could arise if cells release internalized substrates upon death. Make sure this updates the concentration in the region taking into account the volume of that region.

## Resource availability

### Lead contact

Further information and requests for resources should be directed to and will be fulfilled by the lead contact, Daniel Bergman (bergmand@umich.edu).

### Materials availability

This study did not generate new unique reagents.

## Data Availability

The published article includes all code generated or analyzed during this study.

## References

[bib1] Bergman D., Sweis R.F., Pearson A.T., Nazari F., Jackson T.L. (2022). A global method for fast simulations of molecular dynamics in multiscale agent-based models of biological tissues. iScience.

